# Dynamic changes in *In vitro* rumen fermentation, nutrient degradation, and microbial communities of fermentation inoculant-treated licorice stem and leaf silage under different dry matter contents

**DOI:** 10.1371/journal.pone.0353206

**Published:** 2026-07-08

**Authors:** Limin Tang, Haonan Liu, Qifeng Gao, Yuliang Sun, Xinyu Xu, Wenghao Li, Dong Lu, Yulan Ren, Houjun Yu, Tao Jiang

**Affiliations:** 1 College of Animal Science and Technology, Tarim University, Alar, Xinjiang, China; 2 College of Life Science and Technology, Tarim University, Alar, Xinjiang, China; 3 Third Division Animal Husbandry and Fishery Discovery Service Center, Tumushuke, Xinjiang, China; 4 Key Laboratory of Livestock and Forage Resources Utilization around Tarim, Tarim University, Alar, Xinjiang, China; 5 Xinjiang Wensu Dairy Cattle Science and Technology Courtyard, Aksu, Xinjiang, China; Osmania University, INDIA

## Abstract

This study aimed to explore the effects of semi-dry licorice (*Glycyrrhiza uralensis*) stem and leaf silage on dynamic changes in dairy cow *in vitro* rumen fermentation parameters, nutrient degradation rates, and microbial community composition. Silage substrates with four dry matter (DM) contents (45%, 50%, 55%, 60%; L45, L50, L55, L60) were prepared by adjusting moisture with additives, with 5 replicates per group per time point. *In vitro* fermentation lasted 72 h, with cumulative gas production (GP), fermentation parameters, microbial composition, and nutrient degradation rates measured at 0, 3, 12, 24, 72 h. Principal component analysis (PCA) and gray relational analysis (GRA) were used for comprehensive evaluation. Results showed that L55 had significantly higher 72-h GP, microbial crude protein (MCP), and nutrient degradation rates (*P* < 0.05), and lower pH at 3 and 72 h (*P* < 0.05). It also had higher total volatile fatty acid (TVFA) and acetic acid (AA) at 12, 24 h (*P* < 0.05), the highest proportion of unique OTUs at 3, 72 h, and superior α-diversity indices (ACE, Chao1) compared to L45 (P < 0.05). L55 showed dominant early abundances of functional genera (e.g., *Prevotella*) and higher metabolic pathway proportions (*P* < 0.05 vs. L60), ranking first in PCA and GRA. Conclusion: 55% DM in licorice semi-dry silage optimizes *in vitro* rumen fermentation and microbial composition, presenting the best feeding value.

## Introduction

The sustainable development of ruminant livestock farming heavily relies on high-quality roughage resources, while the efficient development of unconventional feed is a key pathway to alleviating the feed supply-demand contradiction and reducing farming costs. Licorice, widely cultivated in China as a medicinal plant, produces stem and leaf by-products rich in crude protein, dietary fiber, and other nutritional components after processing its rhizomes. However, these by-products have long been neglected and wasted, presenting enormous potential for conversion into feed for ruminant animals [1,2]. Silage technology, as a core method for the preservation and quality improvement of roughage, can effectively retain the nutritional value of raw materials and inhibit the proliferation of harmful microorganisms. Among the various factors influencing silage fermentation quality, dry matter content is one of the key factors that directly affects the proliferation of lactic acid bacteria, the production of organic acids, and the subsequent efficiency of ruminal digestion and utilization [3,4].

Numerous studies have confirmed that the dry matter content of silage materials significantly affects fermentation characteristics and nutritional value. In whole-plant rice silage, the increase in dry matter content linearly reduces the yield of organic acids and ruminal digestibility [5]. The dry matter level of alfalfa silage can alter methane emissions and dry matter digestibility in the rumen [6]. The inoculation of silage with Lactobacillus plantarum enhances the yield of digestible nutrients, improves dry matter recovery, and increases the retention rates of protein and soluble carbohydrates, while simultaneously lowering pH and ammonia nitrogen concentrations, thereby improving the fermentation quality of the silage [7,8]. The addition of Lactobacillus buchneri can enhance the aerobic stability of various silages [9]. The addition of lactic acid bacteria composite preparations can further optimize the quality of silage by producing antibacterial substances that inhibit mold growth and secreting esterase to degrade lignocellulose, thereby enhancing the digestibility and utilization rate in the rumen [4]. However, the efficacy of fermentation agents is highly dependent on the dry matter content of the raw materials. Low moisture conditions may limit the activity of the microorganisms, while high moisture levels can easily lead to clostridial fermentation, both of which can reduce the quality of silage [10,11].

The *in vitro* rumen fermentation model has become a core method for evaluating the nutritional value of feed due to its strong controllability and good repeatability. The fermentation parameters (gas production, volatile fatty acids, ammonia nitrogen), nutrient degradation rates, and microbial community composition can comprehensively reflect the dynamic degradation patterns of feed in the rumen [12,13]. Currently, research on the dry matter content of silage has mainly focused on conventional materials such as corn, alfalfa, and grass forage. There is a lack of studies specifically addressing the regulation of dry matter content in the silage of licorice stems and leaves, particularly a systematic analysis of the dynamic changes in fermentation parameters in the rumen, nutrient degradation rates, and microbial community dynamics during the fermentation process. Existing studies have not clarified the dynamic response mechanisms of licorice stem and leaf silage with fermentation agents in the rumen under different dry matter content levels, and the screening of optimal dry matter content lacks scientific basis.

Our group previously found that adding silage additives at a moisture content of 45% yields better results for semi-dry silage of licorice stems and leaves [14]. Based on this, the present study utilized fermentation agents for licorice stems and leaves silage with different dry matter contents (corresponding to moisture contents of 40%, 45%, 50%, and 55%) as substrates. Through *in vitro* rumen fermentation experiments, we systematically measured cumulative gas production, fermentation parameters, nutrient degradation rates, and microbial community composition at 0, 3, 12, 24, and 72 hours. Principal Component Analysis (PCA) and Grey Relational Analysis (GRA) were employed to comprehensively evaluate the effects of dry matter content. The study aims to reveal the dynamic regulatory patterns of dry matter content on the rumen fermentation characteristics of licorice stems and leaves silage. We hypothesize that an appropriate dry matter content can optimize microbial fermentation efficiency and nutrient degradation, providing theoretical support and technical references for the efficient feed utilization of licorice stem and leaf resources.

## Methods

### Experimental materials and silage preparation

Licorice stems and leaves were harvested at the flowering stage from Xin Nong Licorice Pharmaceutical Co., Ltd. in Aral City (40°22′30″ N, 80°03′45″ E). The silage additives were provided by Beijing Precision Animal Nutrition Research Center Co., Ltd. The licorice stems and leaves were chopped into pieces of 2–3 cm and divided into four groups: L45, L50, L55, and L60, with dry matter (DM) contents of 45%, 50%, 55%, and 60%, respectively. The DM content was monitored in real-time, and the determination was conducted using the oven drying method (AOAC, 934.01). If the experimental DM content was not reached, the materials were dried in a cool place, and silage preparation was immediately conducted when close to the target DM content. The raw materials were packed into 2 L plastic jars, compacted to fill completely, with each jar weighing approximately 1.4 kg. The jar openings were sealed with cling film and tape, and the jars were stored in a dark place at room temperature for 60 days before opening, after which air-dried samples were prepared to determine the nutritional components and fermentation quality of the silage feed. The nutritional levels of semi-dry silage made from licorice stems and leaves with different dry matter contents are shown in Table 1 [14].

**Table 1:** The nutritional levels of semi-dry silage of licorice stems and leaves with varying moisture contents (on a Dry Matter Basis).

Groups	DM^1^	CP	EE	NDF	ADF	Ash	WSC
**L45**	45.01	11.92	9.44	49.01	37.11	10.11	1.20
**L50**	49.27	11.46	9.77	46.45	33.80	9.45	1.40
**L55**	54.56	14.00	9.95	40.82	25.92	10.85	2.17
**L60**	58.98	11.43	9.91	47.78	36.12	9.69	1.13

^1^DM = Dry matter; CP = Crude protein; NDF = Neutral detergent fibre; ADF = Acid detergent fibre; WSC = Water-soluble carbohydrates; EE = Ether extract; All data in this table are presented as percentages.

### *In vitro* rumen fermentation system

This experiment was approved by the Tarim University Science and Technology Ethics Committee (Approval No.: PB20250828001; Approval Date: 2025-8-28). Three healthy adult Holstein dairy cows were selected for the study. Rumen fluid was collected from the donor cows using an orogastric tube rumen fluid collector (ANSCITECH, Beijing Jiuyan Technology Co., Ltd., China) before morning feeding. The collected rumen fluid was filtered through four layers of gauze into a thermal flask for use in the laboratory.

Using the method established by Menke [15] et al., *in vitro* gas production and preparation of artificial rumen fluid were conducted. A sample of 200 mg of the feed to be tested was placed into a 100 mL graduated glass syringe, which was sealed with an appropriate amount of petroleum jelly to ensure airtightness and prevent gas leakage. Each sample was repeated five times, with five blank syringes as controls. The rumen fluid was mixed with the artificial rumen fluid at a volume ratio of 1:2, while continuously introducing anaerobic CO_2_ until the solution became colorless, maintaining the temperature at 39 °C throughout this process. Approximately 30 mL of the mixed culture solution was added to each syringe, and the air inside the syringe was expelled before sealing, with the corresponding initial scale value recorded. The culture syringes were then rapidly placed into a preheated (39 °C) water bath shaker (SHZ-B, Jiangsu Jinyi Instrument Technology Co., Ltd.) for incubation. Gas production from each syringe was recorded at 0, 3, 6, 9, 12, 24, 36, 48, and 72 hours.

Using a DaisyII incubator (ANKOM, Shenyang Mulun Technology Co., Ltd., China), *in vitro* cultures were conducted with Holstein cows as rumen fluid donors. F57 filter bags (25μm pore three-dimensional structure) were washed with acetone for 3-5 minutes and then completely dried to remove surfactants that could prevent microbial degradation. Each digestion jar received 1600 mL of mixed buffer solution and 400 mL of rumen fluid. The mixed buffer solution consisted of solution A and solution B in a 1:5 ratio. Solution A contained: potassium dihydrogen phosphate 10 g/L, magnesium sulfate 0.5 g/L, sodium chloride 0.5 g/L, calcium chloride 0.1 g/L, and urea 0.5 g/L; solution B contained: sodium carbonate 15 g/L and sodium sulfide 1 g/L. Prior to the experiment, the stirrer was preheated with water at 39°C. After adding the liquids and samples to each digestion jar, CO2 gas was introduced for 30 seconds, and then the lids were tightly sealed. Samples were taken at 3 h, 12 h, 24 h, and 72 h post-cultivation. After the cultivation period, nylon bags were rinsed with water at 39°C for 5 minutes and then dried in an oven at 65°C until a constant weight was achieved.

### Measurement of indicators

Gas production (GP) is calculated according to the formula GP = a + b(1 – e^-ct^). Here, t represents the fermentation time (h); GP denotes the gas production at time t (mL); a indicates the gas production from the rapidly degradable portion (mL); b refers to the gas production from the slowly degradable portion (mL); c is the gas production rate constant (%/h); and a + b represents the potential gas production (mL). Samples were taken at 0, 3, 12, 24, and 72 hours using a syringe, and immediately placed in ice water to halt fermentation. The pH of the fermentation liquid was measured using a pH meter (FE28-standard, Mettler-Toledo, China). A portion of the collected experimental samples was stored at −20 °C for the determination of ammonia nitrogen (NH_3_-N) using the method reported by Searle [16] et al., volatile fatty acids (VFA) using the method of Yang [17] et al., and microbial crude protein (MCP) using the Coomassie Brilliant Blue method proposed by Bradford [18]. Another portion of the samples was stored at −80 °C for 16S rDNA gene sequencing analysis of rumen bacteria. The determination of dry matter (DM) content in feed and residues was conducted using the oven drying method (AOAC, 934.01). The contents of neutral detergent fiber (NDF) and acid detergent fiber (ADF) were measured using the method developed by Van Soest et al. The degradation rate (%) was calculated using the formula: degradation rate (%) = 100 × (a – b) / a, where ‘a’ represents the nutrient content (g) and ‘b’ represents the nutrient content (g) of the residue after 72 hours of fermentation.

### Microbiome analysis

Genomic DNA was extracted from samples using the TGuide S96 Magnetic Soil/Stool DNA Kit (TGuide Biotechnology Co., Ltd., Beijing) according to the manufacturer’s instructions. The quality and quantity of the extracted DNA were assessed using 1.8% agarose gel electrophoresis, and the concentration and purity of the DNA were measured with a NanoDrop 2000 UV-Vis spectrophotometer (Thermo Scientific, Wilmington, USA). Full-length 16S rRNA genes were amplified using the primers 27F: AGRGTTTGATYNTGGGCTCAG and 1492R: TASGGHTACCTTGTTASGACTT. The PCR products were purified using VAHTS™ DNA Clean Beads (Vazyme, Nanjing, China) and quantified with the Qubit dsDNA HS Assay Kit and Qubit 3.0 Fluorometer (Invitrogen, Thermo Fisher Scientific, Oregon, USA). Sequencing was performed on the PacBio Sequel II platform (Beijing Biomarker Technologies Co., Ltd., Beijing) using the Sequel II Library Kit 2.0.

### Comprehensive analysis based on principal component analysis

The PCA mathematical model is employed to analyze multiple indicators, with calculations referring to the methods of Liu [19] et al.


$$\begin{array}{c} {\text{F}}_{\text{1}}={\text{P}}_{\text{1}\text{1}}{\text{ZE}}_{\text{1}}+{\text{P}}_{\text{21}}{\text{ZE}}_{\text{2}}+\cdots +{\text{P}}_{\text{n1}}{\text{ZE}}_{\text{m}} \end{array}$$

$$\begin{array}{c} {\text{F}}_{\text{2}}={\text{P}}_{\text{1}\text{2}}{\text{ZE}}_{\text{1}}+{\text{P}}_{\text{22}}{\text{ZE}}_{\text{2}}+\cdots +{\text{P}}_{\text{n2}}{\text{ZE}}_{\text{m}} \end{array}$$

$$\begin{array}{c} {\text{F}}_{\text{3}}={\text{P}}_{\text{1}\text{3}}{\text{ZE}}_{\text{1}}+{\text{P}}_{\text{23}}{\text{ZE}}_{\text{2}}+\cdots +{\text{P}}_{\text{n3}}{\text{ZE}}_{\text{m}} \end{array}$$

$$\begin{array}{c} {\text{F}}_{\text{4}}={\text{P}}_{\text{1}\text{4}}{\text{ZE}}_{\text{1}}+{\text{P}}_{\text{24}}{\text{ZE}}_{\text{2}}+\cdots +{\text{P}}_{\text{n4}}{\text{ZE}}_{\text{m}} \end{array}$$

In these equations, p_1i_, p_2i_,  ..., p_ni_ (i=1,n) are the eigenvectors of the covariance matrix Σ of E, and ZE_1_, ZE_2_,  ..., ZE_m_ are the standardized values of the original variables.

### Statistical analysis

The data were initially processed using WPS Office 2021 software. Prior to statistical analysis, the normal distribution of the data was assessed using the Shapiro-Wilk test (*P* > 0.05), which satisfies the prerequisites for parametric testing. A two-way repeated measures ANOVA was conducted using IBM SPSS Statistics 26.0 software, with dry matter content (treatment factor) and *in vitro* fermentation time (time factor) as fixed effects, and the measured indicators as dependent variables. After confirming the homogeneity of variances through Levene’s test (*P* > 0.05), the treatment effects, time effects, and their interactions were analyzed. When the main effects or interactions reached a significant level (*P* < 0.05), Duncan’s multiple comparison method was employed for post hoc testing. Data are presented as means and standard error of the mean (SEM). A *P* value < 0.05 was considered significant, while a *P* value < 0.01 was considered highly significant. The calculations for degradation parameters a, b, and c were performed via nonlinear regression analysis using IBM SPSS Statistics 26.0 software.

The data visualization and correlation analysis, as well as principal component analysis, were conducted using IBM SPSS Statistics 26.0 and Origin 2021. Prior to performing principal component analysis and cluster analysis, the data required normalization. The gray relational analysis was conducted using SPSSAU software, which also necessitated normalization of the data beforehand. For the operational taxonomic unit (OTU) clustering, Usearch (version 10.0) was employed to cluster Reads at a similarity level of 97.0%, resulting in the generation of OTUs. The evaluation of sample Alpha diversity indices was carried out using QIIME2 2020.6 software. Beta diversity calculations were performed using Nonmetric Multidimensional Scaling (NMDS) to analyze the degree of similarity in species diversity among samples. LEfSe Line Discriminant Analysis (LDA) Effect Size analysis first employs the non-parametric factorial Kruskal-Wallis (KW) sum-rank test to detect significantly abundant differential features. Subsequently, LDA is utilized to estimate the effect size of each component (species) abundance on the differential effects. A species correlation network diagram is constructed using R. PICRUSt2 is used to align the feature sequences (16S rRNA) with the reference sequences from the microbial genome database (IMG, Integrated Microbial Genomes) to build an evolutionary tree, thereby predicting the pathway status of the entire community in conjunction with the KEGG pathway information of the genes.

## Results

### *In vitro* rumen fermentation characteristics

As shown in Table 2, with the increase in DM content, the gas production exhibited a trend of first increasing and then decreasing (*P* < 0.01). With the extension of fermentation time, gas production continued to rise (*P* < 0.01). At all time points, the gas production of the L55 group was significantly higher than that of the L45 and L60 groups (*P* < 0.05). The rapid gas production part (A) of the L55 group was significantly higher than that of the other groups (*P* < 0.05), while the slow gas production part (B) of the L60 group was significantly lower than that of the other groups (*P* < 0.05). The potential gas production (A + B) of the L55 and L50 groups was significantly higher than that of the L45 and L60 groups (*P* < 0.05), and the gas production rate constant (C) of the L55 group was significantly lower than that of the L45 and L50 groups.

**Table 2:** The effect of silage from licorice stems and leaves on the gas production during *in vitro* fermentation. (mL).

Items	Groups				SEM	Significance		
	L45	L50	L55	L60		DM content	Time	DM content×Time
**3 h**	11.33^Hb^	13.67^Gb^	19.00^Ga^	11.33^Hb^	0.682	<0.01	<0.01	0.058
**6 h**	19.00^Gbc^	22.00^Fb^	26.00^Fa^	16.67^Gc^				
**9 h**	25.67^Fb^	29.00^Ea^	31.67^Ea^	21.00^Fc^				
**12 h**	30.67^Eb^	34.33^Da^	36.33^Da^	25.00^Ec^				
**24 h**	38.00^Db^	42.67^Ca^	43.67^Ca^	32.00^Dc^				
**36 h**	42.33^Cb^	46.67^Ba^	47.00^BCa^	36.00^Cc^				
**48 h**	44.6^Bb^	49.00^ABa^	50.33^Ba^	40.33^Bc^				
**72 h**	47.33^Ac^	51.33^Ab^	54.67^Aa^	43.00^Ad^				
**A/mL**	2.86^c^	3.83^c^	13.07^a^	7.08^b^				
**B/mL**	43.04^ab^	46.17^a^	39.99^b^	36.12^c^				
**A + B/mL**	45.90^b^	49.99^a^	53.06^a^	43.21^b^				
**C**	0.08^a^	0.08^a^	0.07^b^	0.05^c^				

^1^The values represented by different lowercase letters indicate significant differences in dry matter content under the same *in vitro* fermentation time; whereas the values represented by different uppercase letters indicate significant differences in *in vitro* fermentation time at the same dry matter content (*P* < 0.05).

^2^A = rapid gas production phase; B = slow gas production phase; A + B = potential gas production; C = gas production rate constant.

^3^L45, L50, L55, L60 = The dry matter content is 45%, 50%, 55%, and 60%.

^4^SEM = standard error of means.

Table 3 shows that the content of DM, fermentation time, and their interaction have a highly significant effect on the rumen pH value (*P* < 0.01). As the fermentation time increases, the pH value gradually decreases (*P* < 0.01). The pH values of the L55 group at 3 hours and 72 hours are significantly lower (*P* < 0.05). With the extension of fermentation time, the NH_3_-N content significantly increases (*P* < 0.01), and there is a highly significant interaction between DM content and fermentation time (*P* < 0.01). The NH_3_-N content of the L45 group at 0 hours is significantly higher than that of the L50 and L55 groups (*P* < 0.05), and the NH_3_-N content of the L50 and L55 groups at 72 hours is significantly higher than that of the other groups (*P* < 0.05). The content of DM, fermentation time, and their interaction have a highly significant effect on MCP content (*P* < 0.01). With the extension of fermentation time, the MCP content shows a trend of first increasing and then decreasing (*P* < 0.01). The MCP content of the L50 group at 3 hours is significantly higher than that of the other groups (*P* < 0.05), and the MCP content of the L55 group at 0 hours, 12 hours, 24 hours, and 72 hours is significantly high (*P* < 0.05).

**Table 3:** The effect of silage from licorice stems and leaves on *in vitro* fermentation pH, NH_3_-N, and MCP.

Items	Groups				SEM	Significance		
	L45	L50	L55	L60		DM content	Time	DM content×Time
**pH**								
**0 h**	6.95^A^	6.92^A^	6.94^A^	7.00^A^	0.014	<0.01	<0.01	<0.01
**3 h**	6.88^Bb^	6.86^ABbc^	6.85^Bc^	6.96^Ba^				
**12 h**	6.84^B^	6.83^BC^	6.83^B^	6.82^C^				
**24 h**	6.77^C^	6.81^BC^	6.70^C^	6.74^D^				
**72 h**	6.74^Ca^	6.78^Ca^	6.66^Cb^	6.68^Eb^				
**NH**_**3**_ **-N(mg/L)**								
**0 h**	117.34^Ba^	106.97^Db^	109.98^Cb^	112.15^Bab^	1.813	<0.01	<0.01	<0.01
**3 h**	118.09^B^	113.24^C^	116.42^BC^	116.25^B^				
**12 h**	118.67^B^	114.16^C^	117.75^BC^	119.35^B^				
**24 h**	119.68^B^	119.76^B^	126.03^B^	121.77^B^				
**72 h**	155.53^Ab^	186.21^Aa^	179.44^Aa^	152.36^Ab^				
**MCP(mg/L)**								
**0 h**	53.20^Cb^	52.31^Bb^	57.06^Ca^	51.32^Bb^	1.369	<0.01	<0.01	<0.01
**3 h**	62.10^ABb^	69.63^Aa^	62.60^Bb^	51.71^Bc^				
**12 h**	68.34^Aa^	48.84^BCb^	64.78^ABa^	71.01^Aa^				
**24 h**	58.15^BCb^	47.36^BCc^	68.04^Aa^	69.43^Aa^				
**72 h**	42.80^Db^	43.00^Cb^	62.80^Ba^	41.42^Cb^				

^1^The values represented by different lowercase letters indicate significant differences in dry matter content under the same *in vitro* fermentation time; whereas the values represented by different uppercase letters indicate significant differences in *in vitro* fermentation time at the same dry matter content (*P* < 0.05).

^2^pH = pH value; NH_3_-N = ammonia nitrogen; MCP = microbial crude protein.

^3^L45, L50, L55, L60 = The dry matter content is 45%, 50%, 55%, and 60%.

^4^SEM = standard error of means.

As shown in Table 4, the total volatile fatty acids (TVFA), acetic acid (AA), propionic acid (PA), and butyric acid (BA) content significantly increased with the extension of fermentation time (*P* < 0.01). The AA content in the L55 group at 12 hours and 24 hours was significantly higher (*P* < 0.05), while the PA content in the L55 group at 12 hours and 72 hours was significantly higher than that of other groups (*P* < 0.05). Additionally, the TVFA in the L55 group at 12 hours and 24 hours was significantly higher than that of other groups (*P* < 0.05).

**Table 4:** The effect of silage from licorice stems and leaves on *in vitro* fermentation VFA.

Items	Groups				SEM	Significance		
	L45	L50	L55	L60		DM content	Time	DM content×Time
**AA(mmol/L)**								
**0 h**	17.96^C^	19.8^B^	19.35^C^	15.29^C^	1.455	<0.01	<0.01	0.125
**3 h**	20.01^BC^	21.89^B^	20.17^C^	18.34^C^				
**12 h**	24.00^Bb^	24.68^Bb^	36.34^Ba^	28.44^Bab^				
**24 h**	36.14^Ab^	37.28^Ab^	44.16^Aa^	35.47^Ab^				
**72 h**	41.2^A^	42.10^A^	48.04^A^	40.43^A^				
**PA(mmol/L)**								
**0 h**	7.96^C^	8.18^C^	8.59^C^	7.18^D^	0.527	<0.01	<0.01	0.337
**3 h**	7.98^C^	8.71^C^	8.73^C^	8.52^D^				
**12 h**	10.58^Bb^	10.33^BCb^	14.28^Ba^	11.63^Cb^				
**24 h**	13.32^A^	13.42^AB^	16.56^A^	13.63^B^				
**72 h**	15.00^Ab^	15.13^Ab^	18.04^Aa^	15.42^Ab^				
**BA(mmol/L)**								
**0 h**	1.33^D^	1.72^C^	1.47^D^	1.30^D^	0.124	0.014	<0.01	0.132
**3 h**	1.36^D^	1.91^C^	1.50^D^	1.71^D^				
**12 h**	2.00^C^	2.09^C^	2.77^C^	2.44^C^				
**24 h**	3.19^B^	3.14^B^	3.57^B^	3.01^B^				
**72 h**	4.13^A^	4.18^A^	4.57^A^	4.12^A^				
**TVFA(mmol/L)**								
**0 h**	27.25^D^	29.7^B^	29.41^C^	23.76^C^	1.833	<0.01	<0.01	0.061
**3 h**	29.36^D^	32.51^B^	30.41^C^	28.57^C^				
**12 h**	36.58^Cb^	37.10^Bb^	53.39^Ba^	42.51^Bb^				
**24 h**	52.66^Bb^	53.84^Ab^	64.28^Aa^	52.12^Ab^				
**72 h**	60.33^A^	61.41^A^	70.64^A^	59.96^A^				

^1^The values represented by different lowercase letters indicate significant differences in dry matter content under the same *in vitro* fermentation time; whereas the values represented by different uppercase letters indicate significant differences in *in vitro* fermentation time at the same dry matter content (*P* < 0.05).

^2^pH = pH value; NH_3_-N = ammonia nitrogen; MCP = microbial crude protein.

^3^L45, L50, L55, L60 = The dry matter content is 45%, 50%, 55%, and 60%.

^4^SEM = standard error of means.

As shown in Table 5, with the extension of fermentation time, the *in vitro* dry matter degradation rate (IVDMD), *in vitro* neutral detergent fiber degradation rate (IVNDFD), and *in vitro* acid detergent fiber degradation rate (IVADFD) significantly increased (*P* < 0.01). The L55 group exhibited significantly higher levels of IVDMD, IVNDFD, and IVADFD at 3 hours, 12 hours, 24 hours, and 72 hours compared to the other groups (*P* < 0.05).

**Table 5:** The effect of silage from licorice stems and leaves on the degradation rates of nutrients during *in vitro* fermentation.

Items	Groups				SEM	Significance		
	L45	L50	L55	L60		DM content	Time	DM content×Time
**IVDMD**								
**3 h**	20.01^BC^	21.89^B^	20.17^C^	18.34^C^	0.649	<0.01	<0.01	0.446
**12 h**	24.00^Bb^	24.68^Bb^	36.34^Ba^	28.44^Bab^				
**24 h**	36.14^Ab^	37.28^Ab^	44.16^Aa^	35.47^Ab^				
**72 h**	41.2^A^	42.10^A^	48.04^A^	40.43^A^				
**IVNDFD**								
**3 h**	7.98^C^	8.71^C^	8.73^C^	8.52^D^	0.546	<0.01	<0.01	0.016
**12 h**	10.58^Bb^	10.33^BCb^	14.28^Ba^	11.63^Cb^				
**24 h**	13.32^A^	13.42^AB^	16.56^A^	13.63^B^				
**72 h**	15.00^Ab^	15.13^Ab^	18.04^Aa^	15.42^Ab^				
**IVADFD**								
**3 h**	1.36^D^	1.91^C^	1.50^D^	1.71^D^	0.527	<0.01	<0.01	＜0.01
**12 h**	2.00^C^	2.09^C^	2.77^C^	2.44^C^				
**24 h**	3.19^B^	3.14^B^	3.57^B^	3.01^B^				
**72 h**	4.13^A^	4.18^A^	4.57^A^	4.12^A^				

^1^The values represented by different lowercase letters indicate significant differences in dry matter content under the same *in vitro* fermentation time; whereas the values represented by different uppercase letters indicate significant differences in *in vitro* fermentation time at the same dry matter content (P < 0.05).

^2^IVDMD = *In vitro* dry matter degradation rate; IVNDFD = *In vitro* neutral detergent fiber degradation rate; IVADFD = *In vitro* acid detergent fiber degradation rate.

^3^L45, L50, L55, L60 = The dry matter content is 45%, 50%, 55%, and 60%.

^4^SEM = standard error of means.

### Comprehensive evaluation based on principal component analysis and grey relational analysis

#### Principal component comprehensive analysis.

**Fig 1:**
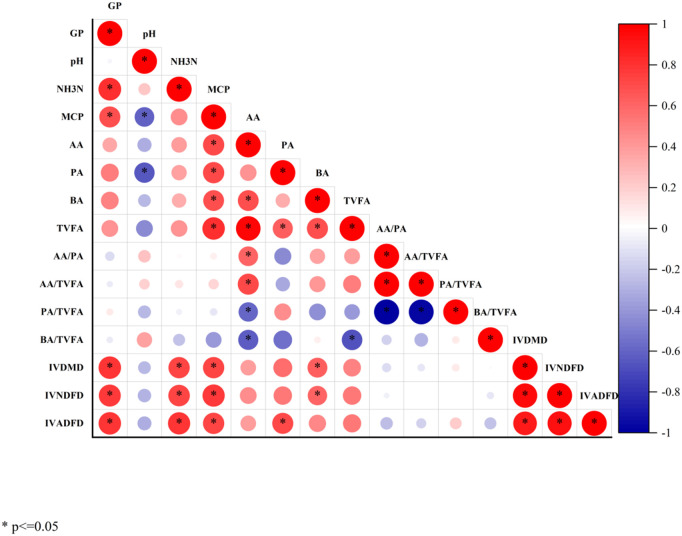
Correlation heatmap of fermentation indicators (P < 0.05). Red indicates a positive corre-lation, and blue indicates a negative correlation, correlation coefficient range: −1 to 1, the deeper the color, the stronger the correlation. Correlation coefficients were calculated using Pearson cor-relation analysis, and Benjamini-Hochberg method was used for multiple comparison correction. The color scale represents the intensity of correlation.

Fig 1 shows that GP is positively correlated with microbial protein (MCP), amino acids (AA), propionic acid (PA), total volatile fatty acids (TVFA), *in vitro* dry matter digestibility (IVDMD), *in vitro* neutral detergent fiber digestibility (IVNDFD), and *in vitro* acid detergent fiber digestibility (IVADFD). Conversely, pH is negatively correlated with MCP, AA, PA, TVFA, IVDMD, IVNDFD, and IVADFD. Additionally, AA, PA, and TVFA are positively correlated with IVDMD, IVNDFD, and IVADFD. MCP is positively correlated with GP, AA, PA, TVFA, IVDMD, IVNDFD, and IVADFD.

From Tables 6 and 7, it can be observed that there are four points with eigenvalues greater than 1, with a cumulative contribution rate of 94.848%. This indicates that the first four principal components can adequately represent the information of the original indicators (gas production, pH, nitrogen metabolism, volatile fatty acids, nutrient degradation rate, etc.). The eigenvalue of the first principal component is 6.951, with significant loadings on positive fermentation indicators such as gas production, nutrient degradation rate, and volatile fatty acids, reflecting the synergistic relationship among these indicators, which is consistent with the results of the previous correlation analysis. The eigenvalues of the second, third, and fourth principal components are 4.055, 2.095, and 1.125, respectively.

**Table 6:** The characteristic values of the principal components and their variance contributions.

	Initial eigenvalue			Extracted sum of squares of load			Sum of squares of rotational loads		
Principak component number	Eigenvalue	Percentage of variance(%)	Cumulative(%)	Eigenvalue	Percentage of variance(%)	Cumulative(%)	Eigenvalue	Percentage of variance(%)	Cumulative(%)
**1**	6.951	46.343	46.343	6.951	46.343	46.343	5.496	36.639	36.639
**2**	4.055	27.037	73.379	4.055	27.037	73.379	4.144	27.627	64.267
**3**	2.095	13.967	87.346	2.095	13.967	87.346	2.544	16.958	81.225
**4**	1.125	7.503	94.848	1.125	7.503	94.848	2.044	13.624	94.848
**5**	0.364	2.424	97.272						
**6**	0.248	1.654	98.926						
**7**	0.074	0.492	99.419						
**8**	0.05	0.335	99.753						
**9**	0.027	0.179	99.933						
**10**	0.009	0.061	99.994						
**11**	0.001	0.006	100						
**12**	3.8E-16	2.6E-15	100						
**13**	1.802E-16	1.201E-15	100						
**14**	5.424E-17	3.616E-16	100						
**15**	−4.60E-17	−3.07E-16	100						

**Table 7:** The principal component loading matrix and characteristic vectors.

	First principal components		Second principal components		Third principal components		Fourth principal components	
Index	Loads	Eigenvectors	Loads	Eigenvectors	Loads	Eigenvectors	Loads	Eigenvectors
**E**_**1**_	0.773	0.293	−0.266	−0.132	0.409	0.283	−0.116	−0.109
**E**_**2**_	−0.421	−0.160	0.243	0.121	0.719	0.497	−0.415	−0.391
**E**_**3**_	0.699	0.265	−0.101	−0.050	0.502	0.347	−0.478	−0.451
^**E**^_**4**_	0.922	0.350	−0.024	−0.012	−0.176	−0.122	0.191	0.180
^**E**^_**5**_	0.769	0.292	0.570	0.283	−0.259	−0.179	−0.066	−0.062
^**E**^_**6**_	0.721	0.273	−0.465	−0.231	−0.455	−0.314	−0.096	−0.091
^**E**^_**7**_	0.727	0.276	0.302	0.150	0.165	0.114	0.511	0.482
^**E**^_**8**_	0.858	0.325	0.360	0.179	−0.326	−0.225	−0.056	−0.053
^**E**^_**9**_	0.114	0.043	0.979	0.486	0.123	0.085	0.014	0.013
^**E**^_**10**_	0.219	0.083	0.969	0.481	0.048	0.033	−0.056	−0.053
^**E**^_**11**_	−0.137	−0.052	−0.966	−0.480	−0.191	−0.132	−0.069	−0.065
^**E**^_**12**_	−0.421	−0.160	−0.197	−0.098	0.653	0.451	0.592	0.558
^**E**^_**13**_	0.842	0.319	−0.293	−0.146	0.337	0.233	0.140	0.132
^**E**^_**14**_	0.872	0.331	−0.219	−0.109	0.304	0.210	0.099	0.093
^**E**^_**15**_	0.858	0.325	−0.389	−0.193	0.187	0.129	−0.098	−0.092

The standardized E_1_, E_2_,  ..., E_15_ are denoted as ZE_1_, ZE_2_,  ..., ZE_15_. Therefore, the principal components are represented as:


$$\begin{array}{cc} {\text{F}}_{\text{1}}=\; & {\text{0}.\text{293ZE}}_{\text{1}}-{\text{0}.\text{160ZE}}_{\text{2}}+{\text{0}.\text{265ZE}}_{\text{3}}+{\text{0}.\text{350ZE}}_{\text{4}}+{\text{0}.\text{292ZE}}_{\text{5}}+{\text{0}.\text{273ZE}}_{\text{6}}+{\text{0}.\text{276ZE}}_{\text{7}}\\ & \;\;+{\text{0}.\text{325ZE}}_{\text{8}}+{\text{0}.\text{043ZE}}_{\text{9}}+{\text{0}.\text{083ZE}}_{\text{10}}-{\text{0}.\text{052ZE}}_{\text{11}}-{\text{0}.\text{160ZE}}_{\text{12}}+{\text{0}.\text{319ZE}}_{\text{13}}+{\text{0}.\text{331ZE}}_{\text{14}}+{\text{0}.\text{325ZE}}_{\text{15}} \end{array}$$

$$\begin{array}{cc} {\text{F}}_{\text{2}}=\; & {-\text{0}.\text{132ZE}}_{\text{1}}+{\text{0}.\text{121ZE}}_{\text{2}}-{\text{0}.\text{050ZE}}_{\text{3}}-{\text{0}.\text{012ZE}}_{\text{4}}+{\text{0}.\text{283ZE}}_{\text{5}}-{\text{0}.\text{231ZE}}_{\text{6}}+{\text{0}.\text{150ZE}}_{\text{7}}\\ & +{\text{0}.\text{179ZE}}_{\text{8}}+{\text{0}.\text{486ZE}}_{\text{9}}+{\text{0}.\text{481ZE}}_{\text{10}}-{\text{0}.\text{480ZE}}_{\text{11}}-{\text{0}.\text{098ZE}}_{\text{12}}-{\text{0}.\text{146ZE}}_{\text{13}}-{\text{0}.\text{109ZE}}_{\text{14}}-{\text{0}.\text{193ZE}}_{\text{15}} \end{array}$$

$$\begin{array}{cc} {\text{F}}_{\text{3}}=\; & \text{0}.\text{283E}\text{1}+{\text{0}.\text{497E}}_{\text{2}}+{\text{0}.\text{347E}}_{\text{3}}-{\text{0}.\text{122E}}_{\text{4}}-{\text{0}.\text{179E}}_{\text{5}}-{\text{0}.\text{314E}}_{\text{6}}+{\text{0}.\text{114E}}_{\text{7}}-{\text{0}.\text{225E}}_{\text{8}}\\ & \;\;+{\text{0}.\text{085E}}_{\text{9}}+{\text{0}.\text{033E}}_{\text{10}}-{\text{0}.\text{132E}}_{\text{11}}+{\text{0}.\text{451E}}_{\text{12}}+{\text{0}.\text{233E}}_{\text{13}}+{\text{0}.\text{210E}}_{\text{14}}+{\text{0}.\text{129E}}_{\text{15}} \end{array}$$

$$\begin{array}{cc} {\text{F}}_{\text{4}}=\; & -{\text{0}.\text{109ZE}}_{\text{1}}-{\text{0}.\text{391ZE}}_{\text{2}}-{\text{0}.\text{451ZE}}_{\text{3}}+{\text{0}.\text{180ZE}}_{\text{4}}-{\text{0}.\text{062ZE}}_{\text{5}}-{\text{0}.\text{091ZE}}_{\text{6}}+{\text{0}.\text{482ZE}}_{\text{7}}\\ & -{\text{0}.\text{053ZE}}_{\text{8}}+{\text{0}.\text{013ZE}}_{\text{9}}-{\text{0}.\text{053ZE}}_{\text{10}}-{\text{0}.\text{065ZE}}_{\text{11}}+{\text{0}.\text{558ZE}}_{\text{12}}+{\text{0}.\text{132ZE}}_{\text{13}}+{\text{0}.\text{093ZE}}_{\text{14}}-{\text{0}.\text{092ZE}}_{\text{15}} \end{array}$$

In the formula, the coefficients are the characteristic vectors of the indicators, while F_1_, F_2_, F_3_, and F_4_ represent the scores of the principal components. The variance contribution rates of the initial eigenvalues are used as the weight coefficients for the first four principal components. This leads to the evaluation model of the indicators, which is the comprehensive score calculated as follows:


$$\begin{array}{c} \text{F}=({\text{0}.\text{46343F}}_{\text{1}}+{\text{0}.\text{27037F}}_{\text{2}}+{\text{0}.\text{13967F}}_{\text{3}}+{\text{0}.\text{07503F}}_{\text{4}})/\text{0}.\text{94848} \end{array}$$

As shown in Table 8, the comprehensive scoring order of the *in vitro* fermentation and degradation indices of licorice stems and leaves with four different dry matter contents is L55 > L50 > L45 > L60.

**Table 8:** Comprehensive score of principal component analysis.

Groups	F_1_	F_2_	F_3_	F_4_	F^1^	Rank
**L45**	−0.873	0.122	−0.257	−0.089	−0.437	3
**L50**	0.714	0.165	−1.357	−0.013	0.195	2
**L55**	1.11	0.026	1.107	0.384	0.743	1
**L60**	−0.95	−0.312	0.507	−0.282	−0.501	4

1 F represents the comprehensive evaluation score calculated by PCA, with the formula: F=(0.38539F_1_ + 0.25485F_2_ + 0.19495F_3_ + 0.06919F_4_)/0.90437.

### Comprehensive analysis using grey relational degree method

As shown in Table 9, the closer the value of the grey relational degree is to 1, the higher the degree of correlation, indicating a stronger alignment of the comprehensive performance of the group with the optimal effect. The L55 group has the highest correlation degree (0.890) among the four groups, corresponding to rank 1; followed by the L50 group (correlation degree 0.770, rank 2), the L60 group (0.760, rank 3), and the L45 group with the lowest correlation degree (0.735, rank 4).

**Table 9:** Comprehensive score of gray correlation degree.

Groups	correlation degree	Rank
**L45**	0.735	4
**L50**	0.770	2
**L55**	0.890	1
**L60**	0.760	3

### Structure characteristics of rumen microbial community

#### OTU distribution.

**Fig 2:**
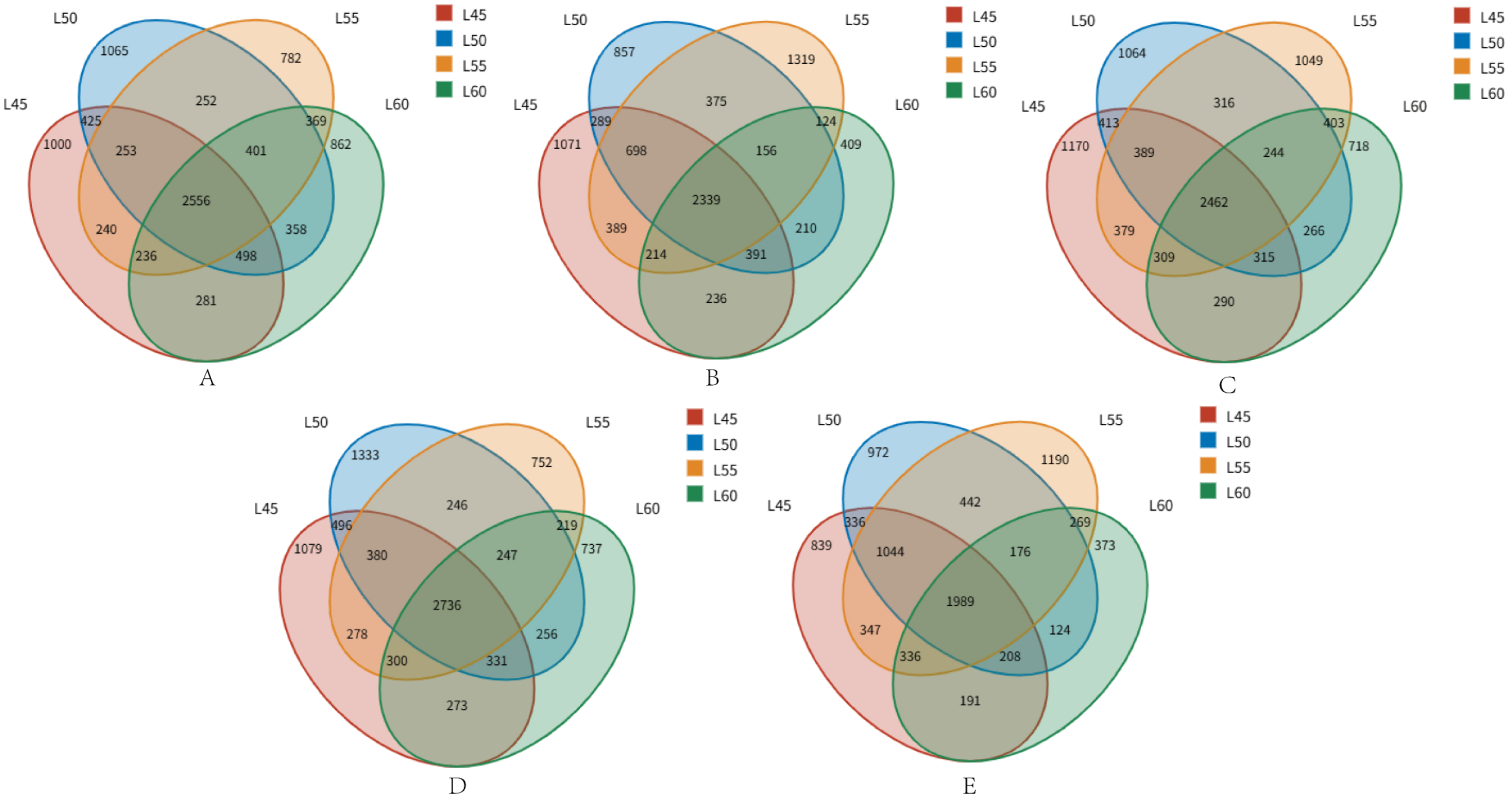
Venn diagram of different fermentation time in each group (in the figure A: 0 hours B: 3 hours C: 12 hours D: 24 hours E: 72 hours).

As shown in Fig 2, at 0 hours, the L50 group exhibited the highest number of OTUs (11.12%), while the L55 group had the lowest (8.16%). At 3 hours, the L55 group showed the highest number of OTUs (14.53%), with the L60 group having the lowest (4.51%). At 12 hours, the L45 group had the highest number of OTUs (11.95%), whereas the L60 group had the lowest (7.34%). At 24 hours, the L45 group again exhibited the highest number of OTUs (12.11%), while the L60 group had the lowest (7.43%). Finally, at 72 hours, the L55 group showed the highest number of OTUs (13.47%), with the L60 group having the lowest (4.22%).

#### α Diversity.

As shown in Table 10, the DM content has a significant impact on the Abundance-based Coverage Estimator (ACE), Chao1 index (Chao1), Simpson index (Simpson), and Shannon-Wiener index (Shannon) (*P* < 0.05). The L55 group showed a significantly higher ACE than the L40 group at both 3 hours and 72 hours (*P* < 0.01), and the Chao1 at 3 hours was also significantly higher than that of the L40 group (*P* < 0.01), indicating a greater number of species in the L55 group. The L45 group exhibited a significantly higher Simpson index than both the L55 and L60 groups at 24 hours (*P* < 0.01), indicating higher species diversity in the L45 group.

**Table 10:** Analysis of α-diversity of rumen microorganisms.

Items	Groups				SEM	Significance		
	L45	L50	L55	L60		DM content	Time	DM content×Time
**ACE**								
**0 h**	6740.67	6699.58	4456.76	5021.31	616.896	<0.01	0.168	0.416
**3 h**	5425.18^a^	4786.39^ab^	6687.15^a^	2373.19^b^				
**12 h**	6385.97	6263.35	5383.06	4255.19				
**24 h**	5844.7	5816.92	4603.43	4347.22				
**72 h**	4577.60^ab^	5581.97^a^	5041.90^a^	2198.79^b^				
**Chao1**								
**0 h**	4683.62	4835.26	3279.9	3860.73	402.841	<0.01	0.236	0.266
**3 h**	4163.88^a^	3709.85^ab^	4771.17^a^	2200.92^b^				
**12 h**	4599.14	4562.57	4001.98	3270.52				
**24 h**	4645.7	4393.25	3711.61	3433.63				
**72 h**	3544.25	4374.85	3940.54	2169.56				
**Simpson**								
**0 h**	0.99	0.99	0.99	0.99	0.001	0.046	0.304	0.219
**3 h**	0.99	0.99	0.99	0.99				
**12 h**	0.99	0.99	0.99	0.99				
**24 h**	1.00^a^	0.99^ab^	0.99^c^	0.99^bc^				
**72 h**	0.99	0.99	0.99	0.99				
**Shannon**								
**0 h**	9.28	9.52	8.68	9.1	0.216	<0.01	0.500	0.779
**3 h**	9.21	9.11	9.32	8.67				
**12 h**	9.44	9.68	8.95	8.82				
**24 h**	9.89	9.67	9.08	8.88				
**72 h**	9.33	9.72	9.48	9.03				

#### β Diversity.

**Fig 3:**
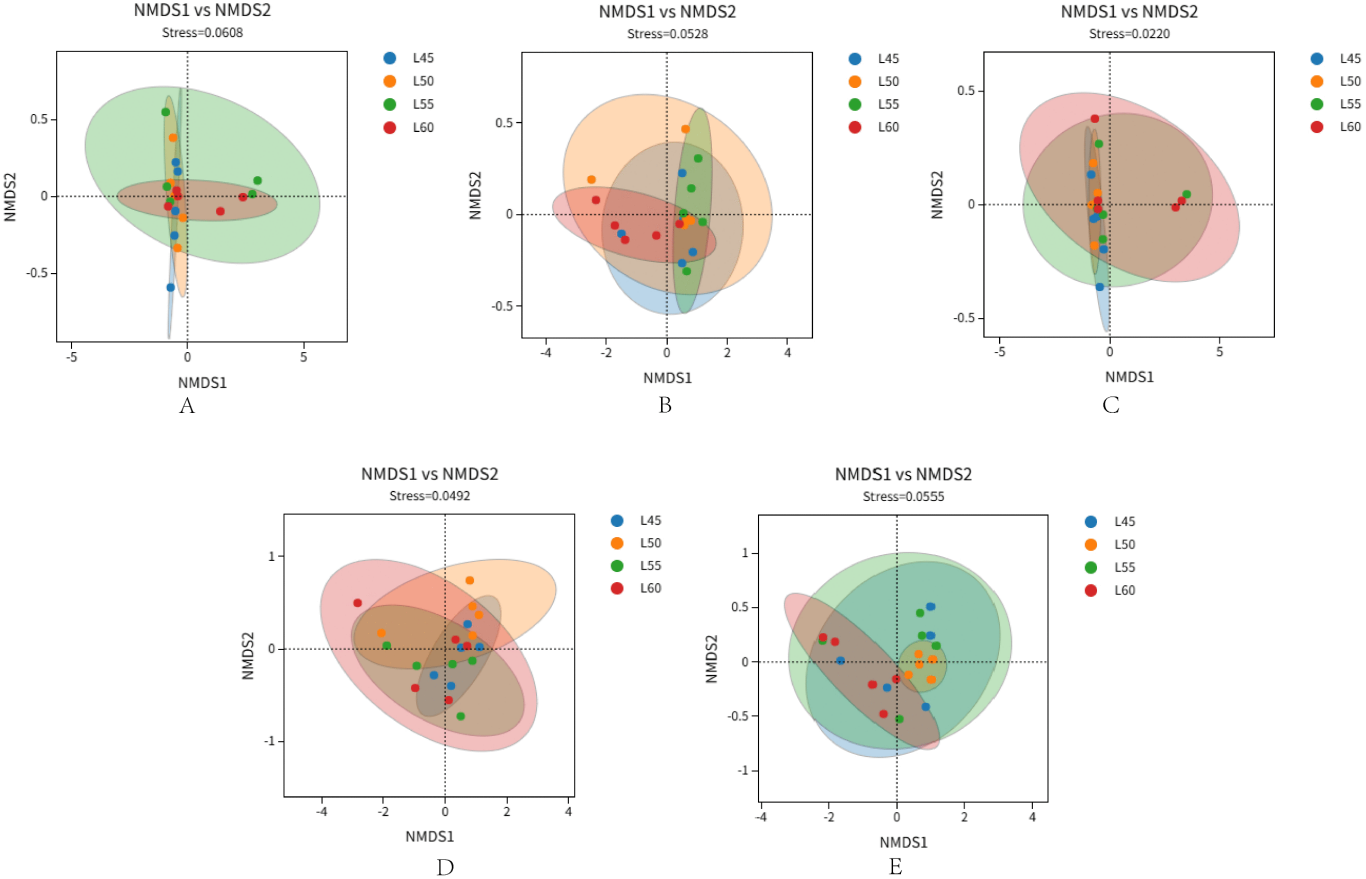
NMDS diagram of each group at different fermentation time (in the Figure A: 0 hours B: 3 hours C: 12 hours D: 24 hours E: 72 hours).

As shown in Fig 3, the differences in the rumen microbial community structure among different DM content groups exhibited a pattern of initial convergence, high differentiation during the mid-phase, re-convergence at the peak phase, and stable differentiation in the later phase with respect to fermentation time. The L55 group demonstrated a high degree of community structure aggregation and stability during key fermentation stages (3h, 72h), which serves as the foundational basis for its optimal fermentation indicators. Conversely, the microbial community structure of the L60 group remained distinct from that of other groups throughout the entire fermentation period.

### Relative abundance at the phylum and genus levels

As shown in Table 11, the dominant phyla in the rumen fermentation fluid for all groups were Firmicutes and Bacteroidota, with relative abundances ranging from 84.58% to 90.90%, 89.09% to 94.02%, 80.97% to 91.66%, and 81.30% to 92.27%. Except for the L60 group at 3 hours, where the dominant phyla were Firmicutes and Proteobacteria. The DM content significantly affected the abundances of Firmicutes, Bacteroidota, Proteobacteria, Planctomycetota, and Cyanobacteria (*P* < 0.05). The fermentation time significantly influenced the abundances of Firmicutes, Bacteroidota, Proteobacteria, and Verrucomicrobiota (*P* < 0.05). At 3 hours, the L60 group showed a significantly higher abundance of Firmicutes compared to the L45 and L55 groups (*P* < 0.01). At 72 hours, the L60 group had a significantly higher abundance of Firmicutes than the other groups (*P* < 0.01). The abundances of Bacteroidota in the L60 group at both 3 hours and 72 hours were significantly lower than those in the other groups (*P* < 0.01). The abundance of Proteobacteria in the L60 group at 72 hours was significantly higher than in the other groups (*P* < 0.01).

**Table 11:** Distribution of phylum-level rumen microorganisms.

Items	Groups				SEM	Significance		
	L45	L50	L55	L60		DM content	Time	DM content×Time
**Firmicutes**								
**0 h**	43.8^C^	51.64	51.74	52.38^C^	4.138	0.024	<0.01	0.383
**3 h**	51.65^BCb^	59.96^ab^	47.64^b^	73.15^BCa^				
**12 h**	51.01^BC^	50.7	56.28	57.49^BC^				
**24 h**	63.86^AB^	61.48	62.92	61.63^AB^				
**72 h**	68.84^Ab^	63.89^b^	69.10^b^	87.26^Aa^				
**Bacteroidota**								
**0 h**	43.69	37.45	29.23	28.92	4.875	<0.01	<0.01	0.438
**3 h**	32.93a	31.35a	43.54a	9.20b				
**12 h**	39.67	42.74	34.26	26.17				
**24 h**	26.27	30.46	21.1	25.55				
**72 h**	22.06^a^	30.13^a^	22.56^a^	5.01^b^				
**Proteobacteria**								
**0 h**	6.13	5.22	12.87	12.77	2.153	<0.01	0.010	0.492
**3 h**	10.49	4.53	3.58	14.29				
**12 h**	5.42	2.54	5.22	12.98				
**24 h**	4.61	3.55	11.37	7.33				
**72 h**	1.77^b^	0.47^b^	1.60^b^	3.30^a^				
**Planctomycetota**								
**0 h**	3.52	2.90	2.97	1.26	0.536	<0.01	0.222	0.863
**3 h**	1.98	1.97	2.96	0.10				
**12 h**	1.79	1.56	2.26	1.23				
**24 h**	1.77	1.67	1.32	1.23				
**72 h**	2.88	1.98	3.26	0.36				
**Cyanobacteria**								
**0 h**	0.71	0.72^A^	1.10	2.71	0.320	<0.01	0.066	0.320
**3 h**	0.73	0.28^B^	0.34	1.89				
**12 h**	0.34	0.27^B^	0.40	0.44				
**24 h**	0.67	0.39^B^	1.36	0.75				
**72 h**	0.91	0.37^B^	0.19	1.13				
**Verrucomicrobiota**								
**0 h**	1.22	1.13	1.24	1.11	0.219	0.156	0.041	0.779
**3 h**	1.26	1.14	1.39	0.37				
**12 h**	0.58	1.02	0.49	0.67				
**24 h**	0.83	0.91	0.51	0.68				
**72 h**	1.47	1.40	1.35	0.70				

Table 12 shows that the DM content significantly affects the abundance of *uncultured_rumen_bacterium*, *Rikenellaceae_RC9_gut_group*, *Christensenellaceae_R_7_group*, *Succinivibrionaceae_UCG_002*, and *Lachnospiraceae_NK3A20_group* (*P* < 0.05). Fermentation time significantly affects the abundance of *Prevotella*, *Succinivibrionaceae_UCG_002*, and *Ruminococcus* (*P* < 0.05). In the 3-hour L55 group, the abundance of *Prevotella* is significantly higher than that of the L60 group (*P* < 0.01). In the 3-hour and 72-hour L60 groups, the abundance of *Rikenellaceae_RC9_gut_group* is significantly lower than that of the other groups (*P* < 0.01). In the 72-hour L60 group, the abundance of *Christensenellaceae_R_7_group* is significantly lower than that of the other groups (*P* < 0.01). In the 3-hour L55 group, the abundance of *Lachnospiraceae_NK3A20_group* is significantly higher than that of the L60 group (*P* < 0.01).

**Table 12:** Distribution of genus-level rumen microorganisms.

Items	Groups				SEM	Significance		
	L45	L50	L55	L60		DM content	Time	DM content×Time
**uncultured_rumen_bacterium**								
**0 h**	6.60	7.18	18.33	13.13	4.307	＜0.01	0.374	0.684
**3 h**	11.18	17.21	6.10	29.48				
**12 h**	6.38	6.28	18.39	18.61				
**24 h**	11.48	13.27	13.45	20.38				
**72 h**	16.41	10.47	19.69	30.78				
**Prevotella**								
**0 h**	26.76^A^	22.59^A^	18.85^AB^	15.62	2.796	0.116	＜0.01	0.239
**3 h**	16.20^BCab^	15.67^ABab^	25.15^Aa^	4.54^b^				
**12 h**	21.83^AB^	22.46^A^	20.16^AB^	14.81				
**24 h**	9.73^C^	10.46^BC^	9.23^BC^	14.42				
**72 h**	1.24^D^	1.75^C^	2.11^C^	0.26				
**Rikenellaceae_RC9_gut_group**								
**0 h**	12.01	10.1^B^	6.55	8.98	1.947	＜0.01	0.210	0.062
**3 h**	12.12^a^	11.32^Ba^	13.84^a^	0.41^b^				
**12 h**	14.37	14.38^B^	8.78	7.88				
**24 h**	9.82	14.07^B^	7.79	5.77				
**72 h**	14.31^a^	21.79^Aa^	14.82^a^	1.68^b^				
**Christensenellaceae_R_7_group**								
**0 h**	14.44	16.19	7.71	10.11	2.014	＜0.01	0.992	0.562
**3 h**	12.92	13.82	15.28	5.89				
**12 h**	16.04	16.10	10.01	8.62				
**24 h**	16.24	13.85	11.75	9.00				
**72 h**	11.22^ab^	19.19^a^	12.64^ab^	5.01^b^				
**Succinivibrionaceae_UCG_002**								
**0 h**	5.60	4.49	11.56	11.14	1.970	0.017	＜0.01	0.441
**3 h**	8.95	2.59	2.98	11.07				
**12 h**	4.75	1.86	3.06	11.26				
**24 h**	3.53	2.71	9.73	5.07				
**72 h**	0.93	0.16	0.88	1.88				
**Lachnospiraceae_NK3A20_group**								
**0 h**	1.62	1.65	1.32	0.76	0.453	<0.01	0.212	0.285
**3 h**	1.91^ab^	2.33^ab^	3.09^a^	0.6^b^				
**12 h**	2.72	2.88	1.87	1.24				
**24 h**	2.33	2.74	1.12	0.96				
**72 h**	2.88	2.47	3.91	0.14				
**Ruminococcus**								
**0 h**	6.52	7.55	7.55^A^	8.69	0.800	0.705	<0.01	0.653
**3 h**	5.92	4.84	5.90^AB^	7.03				
**12 h**	4.39	4.69	3.41^B^	4.87				
**24 h**	4.92	5.31	7.24^A^	3.86				
**72 h**	4.74	4.12	3.81^B^	5.72				

### Differences in microbial community and functional prediction

#### LEfSe differential species.

**Fig 4:**
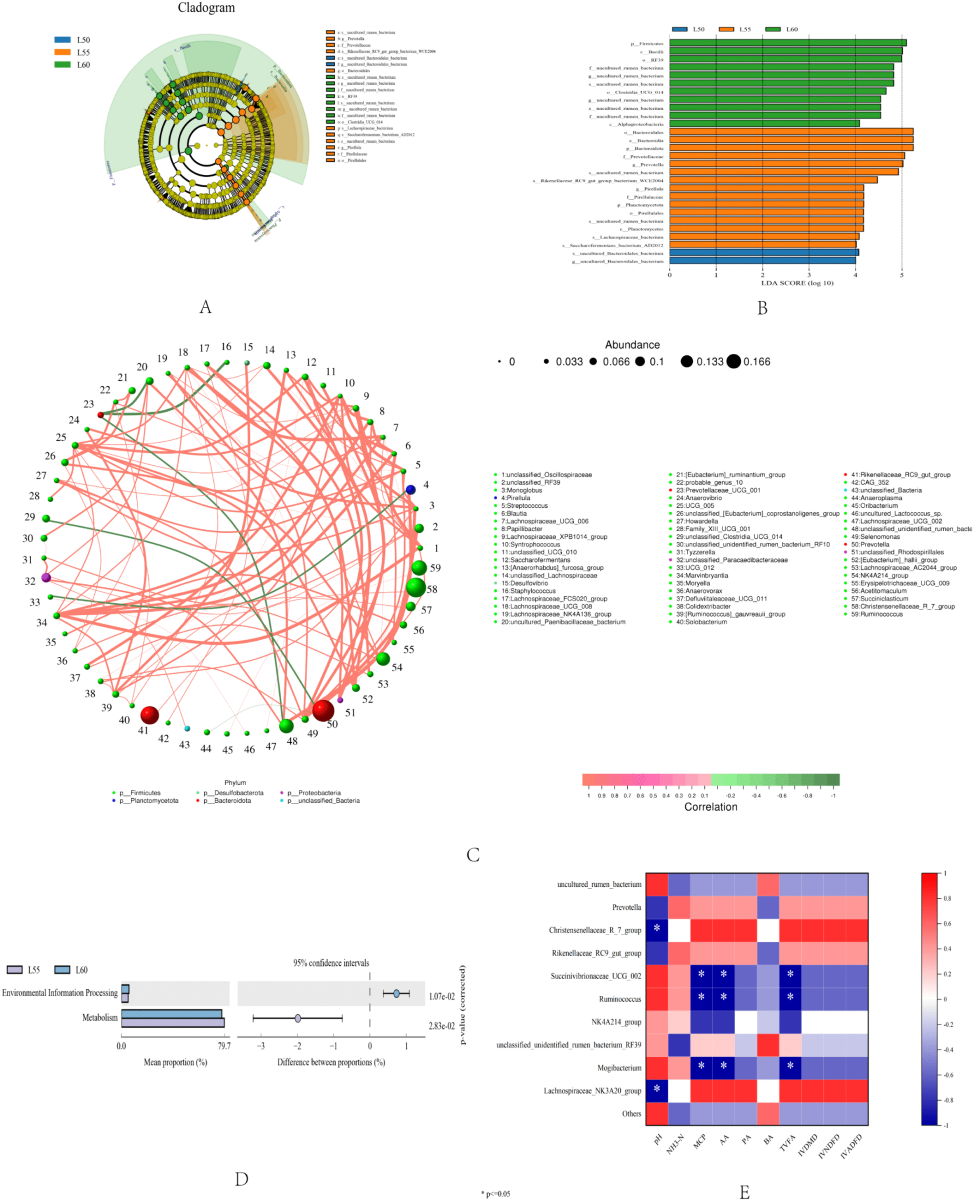
Microbiota difference at 3 h in vitro fermentation, genus-level species network, correlation between fermentation parameters and microbes, and intergroup differential KEGG pathway analysis. A. LEfSe analysis evolutionary branch diagram (phylum to species; the size of the circle is abundance; yellow is not significant, and the others are classified according to the dominant group). B: LDA value distribution histogram (horizontal axis represents the logarithmic score of LDA analysis, where longer bars represent more significant differences). C: network diagram of each species at genus level (circle size is abundance; red is positively correlated and green is negatively correlated). D: The difference analysis of KEGG metabolic pathways between groups (shown in the middle is the difference ratio of functional abundance in the 95% confidence interval, and the rightmost value is p value). E: Correlation diagram of silage fermentation parameters and microorganisms. Red was positively correlated, blue was negatively correlated, * showed significant difference (P < 0.05).

As shown in Fig 4(A, B), In the L50 group, the only differential biomarker identified was the uncultured Bacteroidales bacterium, with an LDA score of ≥4. The differential biomarkers for the L55 group were taxonomic units of the phylum Bacteroidetes (e.g., p_Bacteroidota, c_Bacteroidia, *f_Prevotellaceae*, *g_Prevotella*, etc.), with LDA scores of ≥4, representing the core differential species of the L55 group. The differential biomarkers for the L60 group consisted of multiple taxonomic units from the phylum Firmicutes (e.g., p_Firmicutes, c_Bacilli, o_RF39, etc.), all with LDA scores of ≥4, indicating the core differential species of the L60 group.

#### Species correlation network.

As shown in Fig 4(C), In the figure, the large nodes correspond to functionally core species, with Prevotella, Rikenellaceae_RC9_gut_group, Christensenellaceae_R-7_group, and Ruminococcus identified as the key functional executors during the 3-hour fermentation. The red connections in the network (indicating positive correlations) overwhelmingly dominate, while the green connections (indicating negative correlations) are minimal. This suggests that during the 3-hour fermentation phase, most rumen species exhibit a synergistic relationship, collectively participating in substrate degradation and metabolism.

#### Correlation analysis between microbiota and fermentation indicators.

As shown in Fig 4(D), The core functional bacteria *Prevotella*, *Ruminococcus*, and *Christensenellaceae_R_7_group* showed a positive correlation with the fermentation indicators TVFA, IVDMD, IVNDFD, and IVADFD. In contrast, *Christensenellaceae_R_7_group* and *Lachnospiraceae_NK3A20_group* exhibited a significant negative correlation with pH. Additionally, *Succinivibrionaceae_UCG_002*, *Ruminococcus*, and *Mogibacterium* were significantly negatively correlated with MCP, AA, and TVFA.

#### PICRUSt2 KEGG functional differences.

As shown in Fig 4(E), The proportion of Metabolism functions in the L55 group was significantly higher than that in the L60 group (*P* < 0.05), indicating that this pathway is a major function of the rumen microbiota (supporting substrate degradation, VFA synthesis, and other fermentation processes). Conversely, the proportion of Environmental Information Processing functions in the L60 group was significantly higher than that in the L55 group (*P* < 0.05), suggesting that this pathway is related to the microbiota’s signal perception and adaptation to environmental changes.

## Discussion

The optimal dry matter content for the ensiling of licorice stems and leaves is 55%. Under this condition, the nutritional quality of the substrate is excellent, and the *in vitro* rumen fermentation efficiency is high. The L55 group achieved efficient fermentation by enriching core functional bacteria and enhancing metabolic functions. This study provides a scientific basis for the ensiling preparation of licorice stems and leaves and their application as unconventional feed for ruminants.

Fermentation time and DM × time interaction significantly affected most parameters, indicating that fermentation dynamics varied with substrate composition. Theoretically, the content of fermentable carbohydrates and nitrogen in feed has a significant impact on *in vitro* gas production; the higher the degradation rate of organic matter in the feed, the greater the gas production [20].

In this experiment, there is a significant positive correlation between GP and IVDMD, indicating that the digestible nutrients in the semi-dry silage of different moisture levels of licorice stems and leaves provide ample substrates for microorganisms, thereby improving fermentation. This finding is consistent with the results of Kaplan and Liu et al [21,22]. The higher the nutritional value of the feed, the stronger its fermentability, and the greater the gas production [23]. In this study, the gas production of the L55 group at various time intervals was significantly higher than that of the L45 and L60 groups. The water-soluble carbohydrates (WSC) in the L55 group were higher than those in the other groups, and the substrate for *in vitro* fermentation gas production was primarily provided by WSC [24]. This indicates that the silage from the L55 group can enhance the fermentation characteristics of silage. At this moisture content level, the fibrous components and non-fibrous carbohydrates within the stems and leaves of licorice were well preserved during the semi-dry storage process and did not suffer losses due to excessive respiration. This reflects the utilization by microorganisms and the nutritional value of the feed, which is consistent with the results of the nutritional indicators of the silage in this group. Research by Nsahlai et al. indicates a significant negative correlation between the silage GP of leguminous forages and NDF content. In this experiment, the L55 group exhibited lower NDF content and higher *in vitro* gas production levels, which is consistent with these findings [25].

The normal pH range of the rumen in ruminants is between 5.5 and 7.5 [26]. The concentration of NH_3_-N can reflect the degradation of protein in the feed and the utilization of nitrogen sources by microorganisms. Rumen microorganisms convert nitrogen sources into ammonia, which is synthesized into microbial protein (MPC). The suitable range for NH_3_-N is between 6 and 300 mg/L [27,28]. Microbial crude protein (MCP) typically reflects the growth rate and quantity of microorganisms in the rumen [29]. In this experiment, the pH values of each group at all time points were within the acceptable range, indicating a stable fermentation environment. At 0 hours, the NH_3_-N content of the L45 group was significantly higher than that of the L50 and L55 groups. At 72 hours, the NH_3_-N content of the L50 and L55 groups was significantly higher than that of the other groups. The MCP content of the L55 group was significantly higher at 0, 12, 24, and 72 hours, which may be attributed to the higher CP content in the L55 group, providing more nitrogen sources for microorganisms. This indicates that at this level of dry matter, the rate of nitrogen source release from the licorice stems and leaves is most closely matched with the microbial synthesis rate. As fermentation time extended, the NH_3_-N content of each group gradually increased and remained within an appropriate range. The MCP content of the L45 and L60 groups peaked at 12 hours and was lowest at 72 hours, which is generally consistent with the results of this study [30].

The volatile fatty acid (VFA) content in rumen fluid is a key indicator for evaluating the health status and fermentation state of the rumen, providing 60% to 70% of the energy required for the life activities of ruminants [20,31]. Feed digestibility is one of the important indicators for assessing the nutritional efficiency of diets. For ruminants, the rumen digests most of the dry matter and fiber; the higher the dry matter digestibility, the greater the dry matter intake by ruminants [32,33]. A higher propionate production in the rumen correlates with better production performance in ruminants [34]. In this experiment, the PA content of the L55 group at 12 hours and 72 hours was significantly higher than that of the other groups, indicating that the silage from the L55 group is more beneficial for the digestion and absorption of ruminants. With the extension of fermentation time, the VFA content in each group gradually increased, which is consistent with the results of this study [35,36]. The higher the total volatile fatty acid (TVFA) content in the rumen, the greater the degradation of dry matter in the feed [37,38]. In this experiment, the TVFA levels in the L55 group at 12 hours and 24 hours were significantly higher than those in other groups, and the *in vitro* dry matter digestibility (IVDMD) of the L55 group was significantly greater at all time points compared to differences may be related to the nutritional composition of the silage, soluble carbohydrates, and fiber structure [39]. In this study, the higher IVDMD in the L55 group corresponded to lower neutral detergent fiber (NDF) and acid detergent fiber (ADF) contents, which aligns with the findings of Bao et al [40].

Through the analysis and comparison of the fermentation and degradation indicators of semi-dry ensiled licorice stems and leaves, it is challenging to evaluate their quality based on a single indicator. Therefore, this study employs principal component analysis (PCA) to reduce multiple indicators into several comprehensive indicators, retaining most of the information from the original indicators, which is more accurate than a singular evaluation. The larger the absolute value of the loadings in the principal component matrix, the greater the contribution of the corresponding principal component. The first principal component has a value of zero for GP, MCP, AA, PA, BA, TVFA, and *in vitro* nutrient degradation rates (IVDMD, IVNDFD, IVADFD), all of which are positively correlated, indicating that its essence is a comprehensive factor of fermentation activity and nutrient utilization. A high score in this principal component implies vigorous microbial metabolism, with the increase in GP reflecting an accelerated decomposition rate of substrates by microorganisms. The rise in MCP directly reflects the reproductive efficiency of functional microorganisms, while the accumulation of AA, PA, and TVFA, along with the increases in IVDMD, IVNDFD, and IVADFD, forms a metabolic closed loop of substrate degradation product generation. The efficient degradation of dry matter and fibrous components in the feed provides sufficient carbon sources and energy for microorganisms, thereby driving the synthesis of volatile fatty acids and microbial proteins. This synergy is highly consistent with the earlier correlation heatmap results, where GP, MCP, and VFA exhibit a strong positive correlation with nutrient degradation rates. It confirms that the first principal component can serve as a core indicator for evaluating the intensity of silage fermentation metabolism and nutrient conversion efficiency. Furthermore, it reveals the intrinsic relationships among moisture levels, microbial communities, fermentation indicators, and overall quality. The L55 group optimized the microbial community structure, and the previous results showed that the abundance of Bacteroidota and the diversity of unique OTUs in the L55 group drove the enhancement of fermentation indicators and microbial synergy, ultimately resulting in the optimal overall quality. This finding provides a quantitative basis for moisture regulation in silage feed, allowing for preliminary assessments of silage quality by monitoring core indicators of the first principal component (such as GP, IVDMD, TVFA) and the pH of the third principal component, thereby simplifying the detection process in production practices and facilitating its application in production.

Gray relational analysis is a comprehensive evaluation of multiple fermentation indicators (such as gas production, VFA, degradation rate, etc.). The results further validate that the overall fermentation performance of group L55 is the best among the four groups, which is consistent with the previous conclusions drawn from single fermentation indicators and microbial community structure analysis.

Alpha diversity is typically reflected by core indicators such as ACE, Chao1, Shannon, and Simpson, which indicate species richness and diversity [41,42]. The ACE values for the L55 group at 3 hours and 72 hours were significantly higher than those of the L40 group, while the Chao1 value at 3 hours was also significantly higher for the L55 group. This suggests that the L55 group exhibited the highest microbial species richness under this environmental condition, possibly due to the moderate moisture environment that supports the succession of aerobic residual bacteria while also providing a suitable substrate for anaerobic fermentation microorganisms. The Simpson index for the L45 group at 24 hours was significantly higher than that of the L55 and L60 groups, indicating a higher species diversity in the L45 group. Research has shown that the microbial composition of the rumen in ruminants is primarily composed of Firmicutes, Bacteroidota, and Proteobacteria. In this experiment, the dominant microbial communities at the phylum level in the rumen fermentation fluid across all groups at various time points were also Firmicutes, Bacteroidota, and Proteobacteria, which is consistent with the findings of this study [43–45]. Studies have indicated that in carbohydrate-rich environments, Bacteroidota has a competitive advantage over Firmicutes in terms of carbohydrate degradation [46,47]. The Bacteroidota community contains genes for carbohydrate-active enzymes that facilitate the fermentation of carbohydrates and the production of short-chain fatty acids [48,49]. In this experiment, LEfse analysis revealed that the differential biomarkers in the L55 group were taxonomic units of Bacteroidota, with significantly higher levels of AA and TVFA in the L55 group at 12 and 24 hours, and significantly higher levels of PA in the L55 group at 12 and 72 hours compared to other groups, which aligns with the aforementioned conclusions. *Prevotella* plays a significant role in the metabolism of proteins, starch, and hemicellulose [50]. The *Rikenellaceae RC9 gut group* is involved in the degradation of plant-derived polysaccharides in ruminants, which may facilitate the breakdown of high-fiber feed [51,52]. Furthermore, it has been reported to have a positive correlation with average daily weight gain and feed efficiency [53,54]. In this experiment, the *Prevotella* levels in the L55 group at 3 hours were significantly higher than those in the L60 group. The *Rikenellaceae_RC9_gut_group* levels in the L60 group at both 3 hours and 72 hours were significantly lower than those in the other groups. The L55 group showed significantly higher IVDMD, IVNDFD, and IVADFD contents at 3 hours, 12 hours, 24 hours, and 72 hours compared to the other groups. The proportion of metabolic functions in the L55 group was significantly higher than that in the L60 group, while the proportion of Environmental Information Processing functions in the L60 group was significantly higher than that in the L55 group. This indicates that the microbial community in the L55 group allocates more resources to core metabolic functions, supporting efficient rumen fermentation, whereas the microbial community in the L60 group may allocate resources to environmental adaptation functions due to the stress of a high dry matter environment, resulting in limited metabolic functions and ultimately poor fermentation performance. In this study, the abundance of *Prevotella* was significantly positively correlated with TVFA and PA, while it exhibited a significant negative correlation with rumen pH. This result confirms that a high abundance of *Prevotella* may promote the accumulation of VFA [55] and lower the pH of the rumen. The metabolites of carbohydrate metabolism primarily originate from the degradation of starch and cellulose, which are digested by rumen microbial enzymes into glucose, and then converted into pyruvate through glycolysis; pyruvate is a major precursor for VFA production [56]. The accumulation of TVFA and PA is associated with the rapid and efficient degradation of soluble carbohydrates by these bacteria [57]. Studies have shown that uronic acids constitute a considerable proportion of legume feed, and the H_2_ and CH_4_ produced by Rumen *Lachnospiraceae isolate NK3A20* during growth on galacturonic acid are reduced [58]. In this experiment, the *Lachnospiraceae_NK3A20_group* in the L55 group at 3 hours was significantly higher than that in the L60 group, indicating that the L55 group may produce less H_2_ and CH_4_.

The inherent limitations of *in vitro* experiments indicate that the *in vitro* rumen fermentation system cannot fully replicate the complex physiological environment in vivo (such as rumen motility, host and microbial interactions, and the flow of digesta), which necessitates caution in generalizing the research conclusions and calls for subsequent in vivo validation. This study focuses solely on the synergistic effects of dry matter content and *in vitro* fermentation time, without considering the potential for further multivariable optimization studies involving silage additives (such as lactic acid bacteria and cellulase). The 16S rRNA sequencing can only reflect the microbial community structure and cannot directly verify the metabolic activity of species; thus, the functional predictions made by PICRUSt2 are indirect and require further confirmation through metagenomic and metabolomic studies. The conclusion of this study, which identifies 55% dry matter as the optimal condition for the silage of licorice stems and leaves, provides practical guidance for actual production and offers specific parameters for the silage formulation of this unconventional feed, aiding in alleviating the feed shortage in ruminants and reducing farming costs. Future research could involve *in vivo* experiments to validate the actual feeding effects of the optimal dry matter content (such as milk production performance in dairy cows and growth performance in meat sheep) and explore the synergistic regulatory effects of additives and optimal dry matter content, further enhancing the quality of licorice silage. By integrating metagenomic and metabolomic technologies, a deeper analysis of the molecular mechanisms underlying rumen fermentation of licorice silage can be conducted.

## Conclusions

The dry matter content and *in vitro* fermentation time significantly influence the comprehensive performance of licorice stem and leaf silage, with 55% dry matter content (L55 group) identified as the optimal formulation parameter. This group not only exhibited higher crude protein and soluble carbohydrate content, but also lower fiber components. Grey relational analysis and principal component comprehensive evaluation confirmed that the L55 group had the highest correlation and comprehensive score. Correspondingly, the gas production, volatile fatty acid yield, and substrate degradation rate during *in vitro* rumen fermentation were significantly superior to other groups. At the microbial community level, the L55 group was enriched with core functional genera such as *Prevotella*, *Ruminococcus*, and *Christensenellaceae_R-7_group*. These microbial communities showed a positive correlation with fermentation indicators like TVFA and substrate degradation rate. Functional prediction results further indicated that the metabolic pathway proportion of the L55 group was higher, directly supporting its efficient fermentation metabolic basis. Under the experimental conditions of this study, 55% dry matter content is the optimal condition for licorice stem and leaf silage, demonstrating good feed value. In this study, we only conducted in vitro rumen fermentation experiments. Further in vivo animal feeding trials are recommended to verify the actual feeding effect of licorice stem and leaf silage, and to explore its application value in practical ruminant breeding production.
